# Vaccine-based clinical protection against SARS-CoV-2 infection and the humoral immune response: A 1-year follow-up study of patients with multiple sclerosis receiving ocrelizumab

**DOI:** 10.3389/fimmu.2022.1037214

**Published:** 2022-12-23

**Authors:** Saskia Räuber, Alice Willison, Melanie Korsen, Tristan Kölsche, Kristin S. Golombeck, Benedikt Plaack, Julia Schüller, Niklas Huntemann, Leoni Rolfes, Christina B. Schroeter, Christopher Nelke, Liesa Regner-Nelke, Moritz Förster, Marius Ringelstein, Michael Harry Barnett, Hans-Peter Hartung, Orhan Aktas, Philipp Albrecht, Tobias Ruck, Nico Melzer, Sven G. Meuth, David Kremer

**Affiliations:** ^1^ Department of Neurology, Medical Faculty, Heinrich Heine University Düsseldorf, Düsseldorf, Germany; ^2^ Department of Neurology, Center for Neurology and Neuropsychiatry, LVR-Klinikum, Heinrich-Heine-University Düsseldorf, Düsseldorf, Germany; ^3^ Brain and Mind Center, University of Sydney, Sydney, NSW, Australia; ^4^ Department of Neurology, Medical University of Vienna, Vienna, Austria; ^5^ Department of Neurology, Palacky University, Olomouc, Czechia

**Keywords:** SARS-CoV-2, COVID-19, vaccination, multiple sclerosis, ocrelizumab, B cell response, T cell response

## Abstract

**Introduction:**

Given the varying severity of coronavirus disease 2019 (COVID-19) and the rapid spread of Severe-Acute-Respiratory-Syndrome-Corona-Virus-2 (SARS-CoV-2), vaccine-mediated protection of particularly vulnerable individuals has gained increasing attention during the course of the pandemic.

**Methods:**

We performed a 1-year follow-up study of 51 ocrelizumab-treated patients with multiple sclerosis (OCR-pwMS) who received COVID-19 vaccination in 2021. We retrospectively identified 37 additional OCR-pwMS, 42 pwMS receiving natalizumab, 27 pwMS receiving sphingosine 1-phosphate receptor modulators, 59 pwMS without a disease-modifying therapy, and 61 controls without MS (HC). In OCR-pwMS, anti-SARS-CoV-2(S)-antibody titers were measured prior to the first and after the second, third, and fourth vaccine doses (pv2/3/4). The SARS-CoV-2-specific T cell response was analyzed pv2. SARS-CoV-2 infection status, COVID-19 disease severity, and vaccination-related adverse events were assessed in all pwMS and HC.

**Results:**

We found a pronounced and increasing anti-SARS-CoV-2(S)-antibody response after COVID-19 booster vaccinations in OCR-pwMS (pv2: 30.4%, pv3: 56.5%, and pv4 90.0% were antibody positive). More than one third of OCR-pwMS without detectable antibodies pv2 developed positive antibodies pv3. 23.5% of OCR-pwMS had a confirmed SARS-CoV-2 infection, of which 84.2% were symptomatic. Infection rates were comparable between OCR-pwMS and control groups. None of the pwMS had severe COVID-19. An attenuated humoral immune response was not associated with a higher risk of SARS-CoV-2 infection.

**Discussion:**

Additional COVID-19 vaccinations can boost the humoral immune response in OCR-pwMS and improve clinical protection against COVID-19. Vaccines effectively protect even OCR-pwMS without a detectable COVID-19 specific humoral immune response, indicating compensatory, e.g., T cell-mediated immunological mechanisms.

## 1 Introduction

The negative implications of the coronavirus disease 2019 (COVID-19) pandemic have gained considerable public attention over the past few years. However, the rapid spread of the disease and its apparent varying severity have emphasized the importance of vaccinating vulnerable individuals, including, for instance, multiple sclerosis (MS) patients (pwMS) receiving disease modifying therapies (DMTs). Several studies assessing the humoral and cellular immune responses following severe acute respiratory syndrome coronavirus type 2 (SARS-CoV-2) vaccination found reduced anti-SARS-CoV-2(S)-antibody titers and an impaired SARS-CoV-2-specific T cell response in subgroups of pwMS, depending on the DMT regimen. Specifically, pwMS receiving B cell modulating therapies (Bc-mts) exhibited a reduced humoral immune response, while T cell-mediated immunity was preserved, which was also demonstrated by our own previous study evaluating a cohort of 59 ocrelizumab-treated pwMS (OCR-pwMS) (1–5). Long-term data indicate a weakened and short-lasting humoral response to SARS-CoV-2 vaccination in those patients (6). However, analysis of the anti-SARS-CoV-2-antibody and T cell responses after SARS-CoV-2 booster vaccination has revealed somewhat contradictory results. While some studies reported higher levels of anti-SARS-CoV-2-antibody titers and Maledon et al. reported increased CD4^+^ and CD8^+^ memory T cell responses after booster vaccination (7–11), not all studies observed increased B and T cell responses following the booster vaccine (12). The relevance of those findings with regard to the protection of pwMS from SARS-CoV-2 infection and COVID-19 is particularly important for clinical practice. Such data may enable clinicians to adjust treatment regimens and to devise optimal vaccination strategies. Concerning the general influence of DMTs on immunity, previous studies report opposing results. On the one hand, a lower incidence of COVID-19 infection was reported in pwMS and the choice of DMT was not found to be associated with the risk of COVID-19 (13, 14). On the other hand, treatment with ocrelizumab (OCR), an intravenously administered selective monoclonal anti-CD20-antibody, was linked to a higher probability of COVID-19 and a more severe disease course (15–22). However, another study reported usually mild to moderate disease severity in pwMS receiving ofatumumab, a subcutaneously administered selective monoclonal anti-CD20-antibody (23). Real-world data regarding the probability of SARS-CoV-2 infection and the disease course of COVID-19 in such patients are therefore needed to clarify the efficacy of vaccine-based clinical protection of pwMS receiving DMTs. Moreover, relating humoral and cellular immune responses to the risk of SARS-CoV-2 infection and the development of COVID-19 could yield important information for future vaccination strategies of pwMS receiving DMTs. We here provide a monocentric retrospective study assessing the seroconversion rate following a third and fourth dose of SARS-CoV-2 vaccination in previously seronegative OCR-pwMS. In addition, we analyzed the probability and severity of COVID-19 infection in pwMS receiving OCR compared to (i) pwMS on other DMTs, (ii) pwMS without (w/o) a DMT and (iii) controls without MS (referred to as healthy controls (HC). Furthermore, we evaluated the relevance of anti-SARS-CoV-2(S)-antibodies and a SARS-CoV-2-specific T cell response, as assessed previously (1), for clinical protection from SARS-CoV-2 infection and symptomatic COVID-19. Finally, adverse events (AEs) of SARS-CoV-2 vaccines up to 16 months following the first vaccination were assessed.

## 2 Methods

### 2.1 Study population

**Figure 1:**
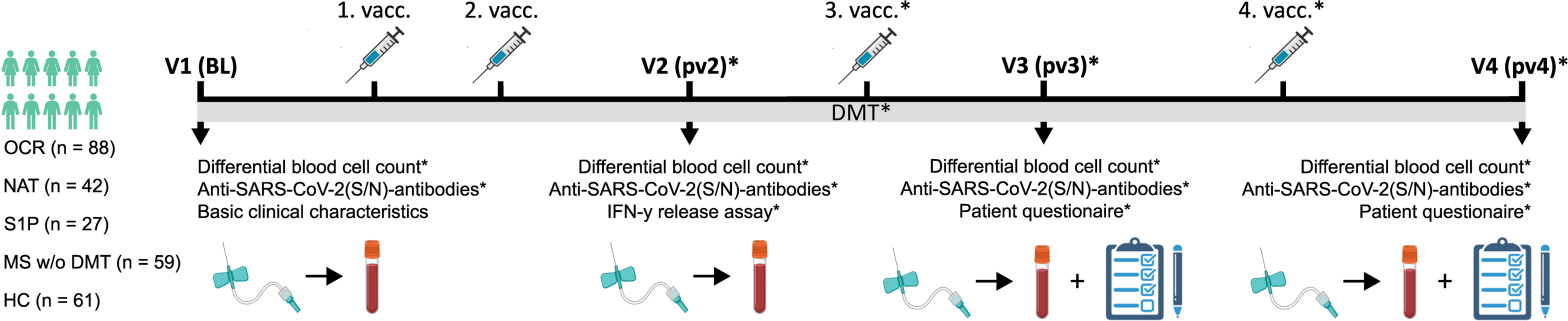
Study design illustrating patient cohorts and the time course of performed analyses. BL, baseline; DMT, disease modifying therapy; HC, controls without MS; IFN, interferon; Ig, immunoglobulin; MS, multiple sclerosis; N, nucleocapsid; NAT, natalizumab; OCR, ocrelizumab; pwMS, patients with MS; pv2/3/4, post second/third/fourth vaccination; S, spike; SARS-CoV-2, severe acute respiratory syndrome; S1P, sphingosine 1-phosphate receptor modulator; V, visit; vacc, vaccination; w/o, without. Created with BioRender.com.

59 OCR-pwMS were included in our initial study analyzing the humoral and cellular immune response after two COVID-19 vaccine doses in 2021 (1). Medical charts were screened to identify patients, who then filled out a patient questionnaire assessing COVID-19 vaccination and infection status. Furthermore, serological parameters were checked to assess anti-SARS-CoV-2(S)-antibody titers after the third and/or fourth vaccine. Overall, 51 of the 59 OCR-pwMS were included in the current study. In order to increase sample size, we additionally reviewed charts of pwMS treated with OCR at the Department of Neurology of the University Hospital Düsseldorf, Germany, between January 1^st^ 2021 and June 15^th^ 2022. Thereby an additional 37 OCR-pwMS who had filled in the patient questionnaire and/or received anti-SARS-CoV-2(S)-antibody testing during clinical routine work-up could be identified. Moreover, 69 pwMS receiving other DMTs (42 natalizumab [NAT-pwMS], 27 sphingosine 1-phosphate modulators [S1P-pwMS]), 59 pwMS without a DMT since the first vaccine [pwMS w/o DMT], and 61 HC who filled out the patient questionnaire were included. All pwMS had been diagnosed by an experienced neurologist in accordance with the 2017 revised McDonald criteria (24). The detailed inclusion and exclusion criteria are summarized in **Table 1**. The study design is shown in **Figure 1**.

**Table 1:** Inclusion and exclusion criteria for MS patients.

**Inclusion criteria** 1. Diagnosis of RRMS, SPMS, or PPMS according to the 2017 revised McDonald criteria 2. Informed consent to participate in the study 3. Age 18 to 75 years old at time of inclusion 4. At least two documented SARS-CoV-2 vaccinations	**Exclusion criteria** 1. Patients who switched DMTs since the first vaccine dose 2. Medical, psychiatric, cognitive, or other conditions that compromise the patient’s ability to understand the patient information and to give informed consent 3. Treatment with mitoxantrone, azathioprine, mycophenolate mofetil, cyclosporine, or methotrexate within the last 5 years 4. Any previous treatment with alemtuzumab, cyclophosphamide, total body irradiation, or bone marrow transplantation 5. Patients who received immunosuppressants for diseases other than MS or who received long-term corticosteroid treatment 6. Patients with verified infection by human immunodeficiency virus or hepatitis C virus 7. Patients with a systemic autoimmune disorder 8. Patients with medical history of COVID-19 infection or positive abs to the SARS-CoV-2 spike protein and/or nucleocapsid protein before the first vaccine dose Regarding patients who received anti-SARS-CoV-2-ab testing and measurement of the SARS-CoV-2-specific T cell response 1. Previous treatment with other B cell modulating therapies (e.g., rituximab, atacicept, belimumab, or ofatumumab) before the start of OCR

ab, antibody, COVID-19, Coronavirus disease 2019, SARS-CoV-2, severe acute respiratory syndrome coronavirus 2.

The study was performed according to the Declaration of Helsinki and was approved by the local Ethics Committee of the Board of Physicians of the Region Nordrhein and of the Heinrich Heine University Düsseldorf, Germany (reference number: 5951R). All patients gave informed consent to participate in the study.

### 2.2 Patient questionnaire

A standardized patient questionnaire was used to assess the number of SARS-CoV-2 vaccinations, vaccination side effects, COVID-19 infection status (asymptomatic SARS-CoV-2 infection confirmed by polymerase chain reaction (PCR) or symptomatic COVID-19 confirmed by either PCR or rapid antigen test) and disease severity (asymptomatic infection, symptomatic infection, hospitalization, intensive care unit (ICU)- treatment) of pwMS receiving different DMTs, pwMS w/o DMT, and HC.

### 2.3 Routine blood analysis

Routine blood tests were performed in the central laboratory of the University Hospital Düsseldorf. Flow cytometry was used to analyze leukocyte subsets (CD19^+^ B cells, CD3^+^ T cells, CD3^+^CD4^+^ T helper cells, CD3^+^CD8^+^ cytotoxic T cells, and CD56^+^CD16^+^ NK cells). Blood samples were prepared using the BD Multitest 6-Color TBNK Reagent (BD Biosciences) according to the manufacturer’s instructions. Data acquisition and analysis was performed with a BD Canto (BD Biosciences).

### 2.4 Measurement of anti-SARS-CoV-2-antibodies

Anti-SARS-CoV-2-antibody-analysis in peripheral blood (PB) was performed as a part of routine clinical practice. Immunoassays for the quantitative assessment of antibodies to the SARS-CoV-2 spike (S) protein and nucleocapsid (N) protein (Elecsys Anti-SARS-CoV-2, Roche Diagnostics) were performed according to the manufacturer’s instructions. A titer of ≥ 0.8 (anti-SARS-CoV-2(S)-antibodies) and ≥ 1.0 (anti-SARS-CoV-2(N)-antibodies) was considered positive. Analysis was performed prior to the first COVID-19 vaccination, after the second (median of (~) 4.1 [range: 2.6 - 16.6] weeks), after the third (~ 9.0 [range: 3.6 - 33.7] weeks), and after the fourth vaccine (~ 7.0 [range: 2.7 - 12.6] weeks). Data from the analysis of anti-SARS-CoV-2-antibodies after two vaccination doses have partly been published before (1). Seroconversion after the third and/or fourth vaccine dose was assessed in previously antibody-negative OCR-pwMS after two vaccines. In addition, the anti-SARS-CoV-2(S)-antibody response was correlated with the probability and severity of COVID-19 infection as assessed by the patient questionnaire.

### 2.5 Quantification of T cell response to SARS-CoV-2

The SARS-CoV-2-specific T cell response in PB assessed by the SARS-CoV-2 Interferon-gamma Release Assay (IGRA; Euroimmun) was measured ~4 weeks after the second dose of COVID-19 vaccination, as previously described (1). The recombinant S1 subunit of the SARS-CoV-2 spike protein served as antigen. The SARS-CoV-2-specific T cell response was correlated with the probability and severity of COVID-19 infection as assessed by the patient questionnaire.

### 2.6 Data analysis

‘GraphPad Prism’ (version 9.0.0) was used to perform data analysis and visualization. Data are shown as the median with the range. The D’Agostino & Pearson test was used to test for normality. The Spearman correlation coefficient was used for correlation analysis. In the case of continuous variables, differences between two groups were assessed using the Mann-Whitney U test when comparing two groups or the Kruskal-Wallis test with Dunn test for multiple comparisons. For binary data, Fisher’s exact test (two groups) or Chi-square test (more than two groups) was used. A p-value of ≤ 0.05 was considered significant.

## 3 Results

### 3.1 Baseline cohort characteristics

In total, 152 relapsing-remitting MS (RRMS) (70.4%), 22 secondary progressive MS (SPMS) (10.2%), and 41 primary progressive MS (PPMS) (19.0%) patients were included in the study. Unfortunately, for one patient no information on the MS disease course was available. 88 pwMS received OCR, 42 NAT, 27 S1P, and 59 no DMT since the first vaccine, of which 42 were treatment-naïve. In addition, 61 HC were included (**Figure 1**). Basic cohort characteristics are summarized in **Table 2** and **Supplementary Table 1**.

**Table 2:** - Demographics and basic disease characteristics.

	OCR	NAT	S1P	W/o DMT	HC
**Patients (number [%])**	88 [40.7]	42 [19.5]	27 [12.5]	59 [27.3]	61
**Age (y)** **(median [range])**	50 [20 - 68]	40 [19 - 71]	48 [18 - 62]	49 [22 - 65]	38 [21 - 73]
**Sex (% female)**	54.0	73.8	70.4	69.5	57.4
**Disease duration (y) (median [range])**	11 [2-38]	15 [0 - 29]	17 [1 - 41]	6 [0 - 10]	–
**Duration of current treatment (y)** **(median [range])**	3 [1 - 9]	9 [0 - 21]	2 [0 - 11]	–	–
**Number of previous therapies** **(median [range])**	1 [0 - 4]	1 [0 - 4]	1 [0 - 4]	0 [0 - 3]	–

DMT, disease modifying therapy; HC, controls without MS; MS, multiple sclerosis; NAT, natalizumab; OCR, ocrelizumab; S1P, Sphingosine-1-Phosphate Modulator; w/o, without; Y, years.

### 3.2 Additional COVID-19 vaccinations can boost the humoral immune response in pwMS treated with ocrelizumab

**Figure 2:**
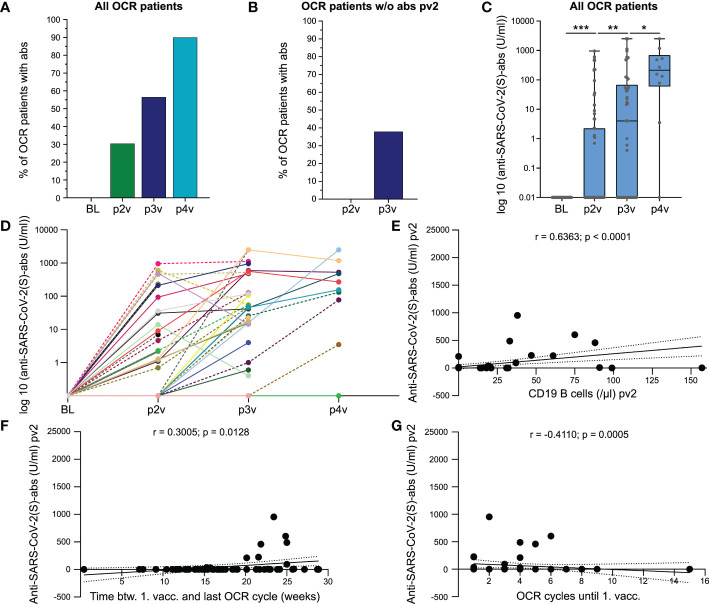
COVID-19 booster vaccinations can increase humoral immune response in MS patients treated with ocrelizumab. **(A)** Percentage of OCR-pwMS with positive anti-SARS-CoV-2(S)-antibodies post second, third, and fourth vaccination; **(B)** Seroconversion after the third vaccine shot in previously antibody negative OCR-pwMS; **(C)** Anti-SARS-CoV-2(S)-antibody titers post second, third, and fourth vaccination. Values < 0.4 U/ml are depicted on the x-axis; **(D)** Individual anti-SARS-CoV-2(S)-antibody titers at BL, pv2, pv3 and pv4. Connection lines between the time points serve as orientation and do not reflect the anti-SARS-CoV-2(S)-antibody level between two time points; Correlation of anti-SARS-CoV-2(S)-antibodies with CD19 B cell count pv2 **(E)**, time between first vaccination and last OCR cycle **(F)**, and number of previous OCR cycles **(G)**. Correlation analyses were performed with the Spearman correlation coefficient. The area in-between the dotted lines shows the 95% confidence interval. *Abs, antibodies; BL, baseline; btw, between; COVID-19, coronavirus disease 2019*; OCR, ocrelizumab; pv2/3/4, post second/third/fourth vaccination; S, spike; SARS-CoV-2, severe acute respiratory syndrome; w/o, without; vacc., vaccination. *p ≤ 0.05, **p ≤ 0.01, ***p ≤ 0.001.

An attenuated anti-SARS-CoV-2(S)-antibody-response was detected in pwMS receiving Bc-mts (1, 4). We measured anti-SARS-CoV-2(S)-antibodies after the second (pv2, n = 69), third (pv3, n = 46), and fourth dose (pv4, n = 10) of COVID-19 vaccine as well as peripheral B cell counts. 30.4% (21/69) of OCR-pwMS had positive anti-SARS-CoV-2(S)-antibodies after two vaccinations, 56.5% (26/46) after three, and 90.0% (9/10) after four (**Figure 2A**). 37.9% (11/29) of OCR-pwMS with no detectable anti-SARS-CoV-2(S)-antibodies after two vaccine doses had a positive antibody response after the third vaccination (**Figure 2B**). A significant increase in anti-SARS-CoV-2(S)-antibody-levels could be detected after the third vaccine dose compared to anti-SARS-CoV-2(S)-antibody-levels following the second vaccination (pv2: 0 [0 - 954] U/ml; pv3: 4 [0 - 2500] U/ml; p = 0.0019). The fourth vaccine dose provided a further significant boost of the humoral immune response (pv4: 213 [0 - 2500] U/ml; p = 0.05; **Figures 2C, D**). As SARS-CoV-2 infection impacts antibody titers, we calculated the time between SARS-CoV-2 infection and antibody testing after the second, third, and fourth vaccine dose. Blood samples pv2 were taken prior to SARS-CoV-2 infection in all but one patient in whom anti-SARS-CoV-2(S)-antibodies were measured 6.86 weeks post infection. Four patients with available anti-SARS-CoV-2(S)-antibodies pv3 were previously infected with SARS-CoV-2 a median of 3.58 [1.29 - 31.29] weeks prior to pv3. Only one patient who received anti-SARS-CoV-2(S)-antibody testing pv4 was previously infected with SARS-CoV-2 (5 weeks prior to pv4). When excluding those patients from the analysis to rule out confounding of anti-SARS-CoV-2(S)-antibody titers by SARS-CoV-2 infection, differences between pv2 and BL as well as between pv2 and pv3 remained significant. Given the low number of patients with available anti-SARS-CoV-2(S)-antibodies pv4, the increase in antibody titers between pv3 and pv4 was no longer significant (data not shown).

In order to rule out relevant confounding by differences in time between analysis of anti-SARS-CoV-2(S)-antibody titers and last vaccination, we compared the median time between antibody negative (ab^-^) and antibody positive patients (ab^+^). We found that the time between the second vaccination and antibody testing was comparable between the two groups (ab^–^patients pv2: 4.1 [2.6 - 16.6] weeks; ab^+^-patients pv2: 4.4 [2.7 - 8.1] weeks; p = 0.5809). Time between anti-SARS-CoV-2(S)-antibody testing and third vaccination tended to be longer in the ab^-^ group; however, differences were not significant (ab^–^patients pv3: 10.3 [4.0 - 33.7] weeks; ab^+^-patients pv3: 6.1 [3.6 - 22.0] weeks; p = 0.1590). At pv4, only one patient had a negative anti-SARS-CoV-2(S)-antibody titer. Analysis was performed 12.6 weeks after the fourth vaccine dose compared to 6.0 [2.7 - 12.6] weeks in ab^+^-patients. In addition, no significant differences in anti-SARS-CoV-2(S)-antibody titers could be observed between female and male OCR-pwMS or between RRMS and PPMS patients (**Supplementary Figures 1A, B**). Correlation analysis revealed a positive correlation between anti-SARS-CoV-2(S)-antibody titers and the peripheral B cell count pv2 (r = 0.6363; p < 0.0001; **Figure 2E**). Furthermore, anti-SARS-CoV-2(S)-antibody levels pv2 positively correlated with the time between the first vaccination and the last OCR cycle (r = 0.3005; p = 0.0128; **Figure 2F**). In addition, anti-SARS-CoV-2(S)-antibody titers pv2 negatively correlated with the number of previous OCR cycles (r = -0.4110; p = 0.0005; **Figure 2G**). No correlation could be found between anti-SARS-CoV-2(S)-antibodies and age, disease duration, or number of previous DMTs (**Supplementary Figures 1 C–E**). Taken together, we found that while the humoral immune response to COVID-19 vaccines is impaired in pwMS receiving Bc-mts, additional COVID-19 vaccines can significantly boost the humoral immune response.

### 3.3 Effective vaccine-based clinical protection of OCR-pwMS

**Figure 3:**
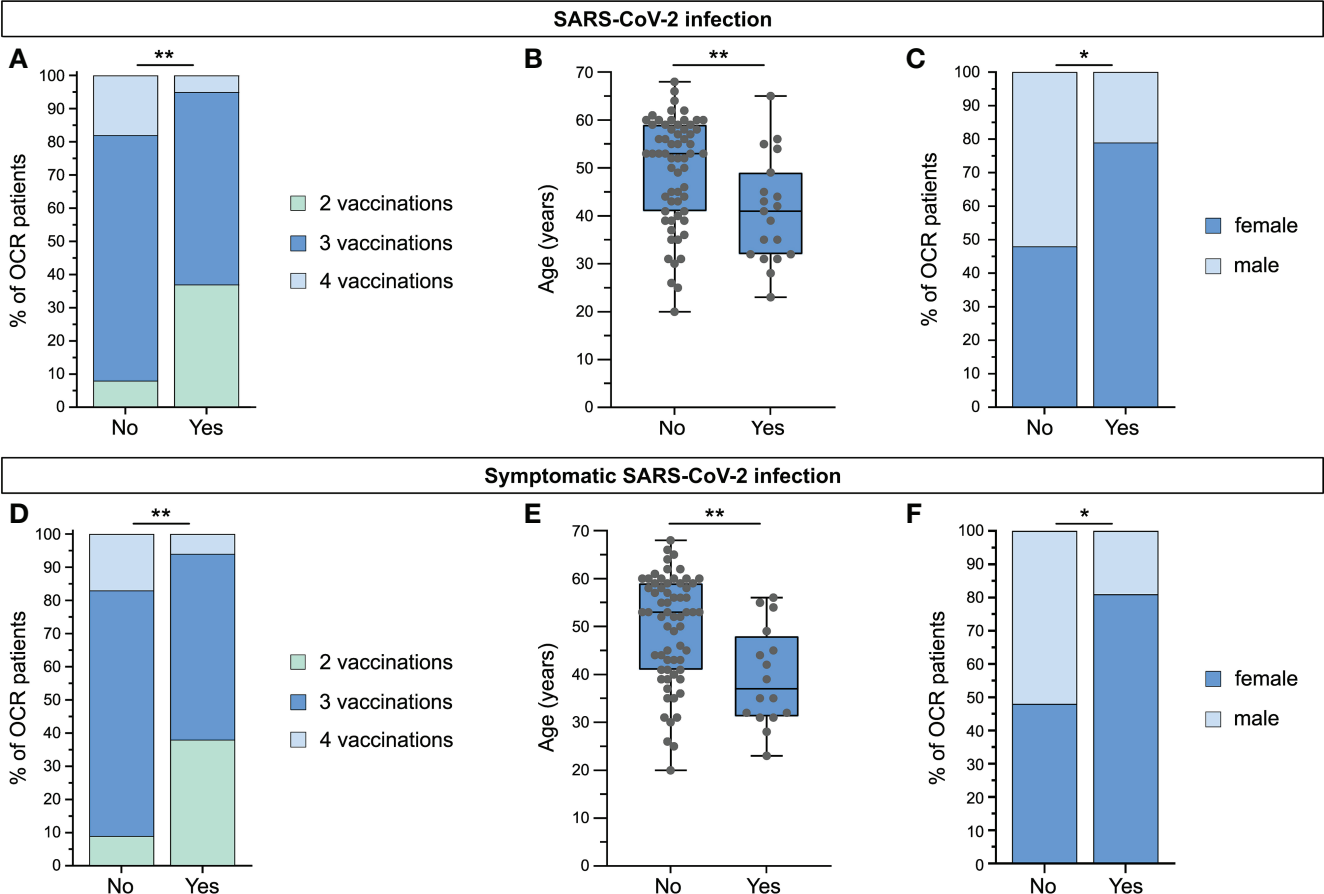
Vaccine-based clinical protection of MS patients receiving ocrelizumab. **(A)** Differences in vaccine doses between OCR-pwMS infected with SARS-CoV-2 and non-infected patients; **(B)** Comparison of age between SARS-CoV-2-infected OCR-pwMS and non-infected patients. **(C)** Differences in sex between non-infected and infected OCR-pwMS; **(D)** Comparison of vaccine doses between OCR-pwMS with and without symptomatic SARS-CoV-2 infection; **(E)** Differences in age between OCR-pwMS with and without symptomatic SARS-CoV-2 infection; **(F)** Differences in sex between OCR-pwMS with and without symptomatic SARS-CoV-2 infection. *p ≤ 0.05, **p ≤ 0.01. OCR, ocrelizumab; SARS-CoV-2, severe acute respiratory syndrome.

Real-world data on vaccine-based clinical protection of pwMS from SARS-CoV-2 infection and COVID-19 are of particular importance for clinical practice, e.g., for the potential adjustment of treatment regimens and optimal vaccination strategies. Of relevance to this study, Bc-mts have been linked to a higher probability of COVID-19 infection and a more severe disease course (15–21). Therefore, we assessed the SARS-CoV-2 infection status and COVID-19 disease course in a cohort of 81 OCR-pwMS. In total, 23.5% (19/81) of patients had been infected with SARS-CoV-2, of which 84.2% (16/19) had symptomatic COVID-19. The majority of OCR-pwMS was infected early in 2022 when Omicron was the prevailing variant in Germany based on data by the Robert Koch Institute (25). None of the OCR-pwMS required hospitalization or ICU-treatment. Patients who had not been infected with SARS-CoV-2 had received significantly more vaccines compared to patients who were (**Figure 3A**). Interestingly, infected patients tended to be younger than non-infected patients (**Figure 3B**), and more female than male OCR-pwMS were infected with SARS-CoV-2 (**Figure 3C**). The same results were found for symptomatic COVID-19 (**Figures 3D–F**). As chronic diseases are associated with a higher prevalence of COVID-19 and a more severe disease course (26, 27), we analyzed differences in SARS-CoV-2 infection and symptomatic COVID-19 between patients with and without comorbidities (comorbidities in general, hypertension, and diabetes) in our patient cohort. No relevant differences could be found (**Supplementary Figures 2A–C**). Moreover, infection status did not differ significantly between RRMS and PPMS patients (**Supplementary Figure 2D**). As previous studies found an attenuated humoral immune response in pwMS receiving Bc-mts (1, 2, 4), we assessed the impact of the humoral and cellular immune response on the clinical outcome. The percentage of OCR-pwMS with positive anti-SARS-CoV-2(S)-antibody titers (non-infected patients: pv2; infected patients: at last antibody testing prior to infection (pv2 or pv3, depending on the time of infection)) was comparable between non-infected and infected patients as well as between patients with and without symptomatic COVID-19 (**Supplementary Figure 2E**). Likewise, the percentage of infected patients was not increased among OCR-pwMS without anti-SARS-CoV-2(S)-antibodies compared to patients with positive antibody titers (**Supplementary Figure 2F**). In addition, no significant differences in anti-SARS-CoV-2(S)-antibody titers (non-infected patients: pv2; infected patients: last antibody testing prior to infection (pv2 or pv3, depending on the time of infection)) were detected between groups (**Supplementary Figure 2G**). Furthermore, interferon-γ release by SARS-CoV-2-specific T cells was not significantly different between non-infected and infected patients as well as between patients with and without symptomatic COVID-19 (**Supplementary Figure 2H**). As there is evidence of a compensatory T cell response in seronegative patients mediating clinical protection, we compared the probability of SARS-CoV-2 infection and symptomatic COVID-19 between seronegative patients with a detectable SARS-CoV-2-specific T cell response (abs^-^IGRA^+^) and seropositive patients with a SARS-CoV-2-specific T cell response (abs^+^IGRA^+^). The percentage of SARS-CoV-2 positive patients was comparable between the two groups (abs^-^IGRA^+^: 22.6% (7/31) versus abs^+^IGRA^+^: 20.0% (2/10); p = 0.8571). Likewise, the percentage of patients suffering from symptomatic COVID-19 did not markedly differ between groups (abs^-^IGRA^+^: 16.1% (5/31) versus abs^+^IGRA^+^: 20.0% (2/10); p > 0.9999) (**Supplementary Figure 1I**). Around one third of the abs^-^IGRA^+^-OCR patients developed positive anti-SARS-CoV-2(S)-antibodies pv3. Overall, SARS-CoV-2 vaccines mediate effective clinical protection of OCR-pwMS. Attenuated humoral immune response was not associated with a higher risk of SARS-CoV-2 infection or a more severe disease course, supporting the relevance of T cell-mediated immunity for clinical protection.

### 3.4 Effective clinical protection and satisfactory safety profile of COVID-19 vaccines among pwMS irrespective of treatment regimen

**Figure 4:**
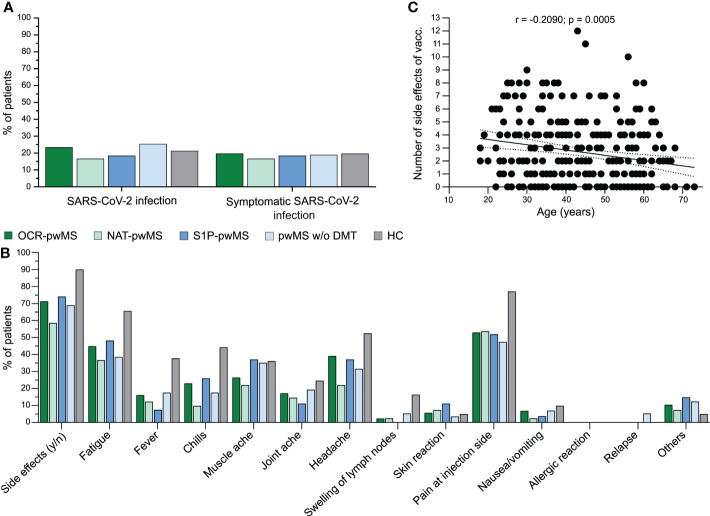
Effective vaccine-based clinical protection of MS patients irrespective of treatment regimen and satisfactory safety profile. **(A)** Comparison of probability of asymptomatic and symptomatic SARS-CoV-2 infection between OCR-pwMS, NAT-pwMS, S1P-pwMS, pwMS w/o DMT, and HC; **(B)** Comparison of side effects between OCR-pwMS, NAT-pwMS, S1P-pwMS, pwMS w/o DMT, and HC; **(C)** Correlation of number of side effects with age. Correlation analysis was performed with the Spearman correlation coefficient. The area in-between the dotted lines shows the 95% confidence interval. OCR, ocrelizumab; HC, controls without MS; MS, multiple sclerosis; n, no; NAT, natalizumab; SARS-CoV-2, severe acute respiratory syndrome; pwMS, patients with MS; S1P, sphingosine 1-phosphate receptor modulator; vacc., vaccination; w/o, without; y, yes.

In order to address the question whether OCR-pwMS are at an increased risk of SARS-CoV-2 infection compared to pwMS receiving other DMTs, pwMS without DMTs, and HC, we compared the percentage of SARS-CoV-2-infected individuals and the COVID-19 disease course between groups. The probability of SARS-CoV-2 infection and symptomatic COVID-19 was comparable between OCR-pwMS, NAT-pwMS, S1P-pwMS, pwMS w/o DMT, and HC (**Figure 4A**). Of note, none of the 216 pwMS was hospitalized or required ICU treatment. Furthermore, we analyzed the safety profile of SARS-CoV-2 vaccines in pwMS on different DMTs and pwMS w/o DMTs compared to HC. Overall, side effects were mild and less pronounced in pwMS compared to HC (**Figure 4B**). Correlation analysis revealed a negative correlation between the number of side effects and age (r = -0.2090; p = 0.0005; **Figure 4C**). Three pwMS w/o DMTs reported symptoms meeting the criteria for a relapse in close temporal association with COVID-19 vaccination, two of them being first diagnosed with MS at this time. No relapse occurred among pwMS on DMTs (**Figure 4B**). No severe side effects were noted for pwMS and HC pointing towards a favorable safety profile of COVID-19 vaccines. Taken together, our results indicate that pwMS w/o DMT but also pwMS receiving OCR, NAT, and S1P are not at an increased risk for SARS-CoV-2 infection and severe disease course. The safety profile of COVID-19 vaccines is favorable among pwMS with and without DMTs as well as HC.

## 4 Discussion

Previous studies reported an attenuated humoral and/or T cellular immune response to SARS-CoV-2 vaccination in pwMS receiving different DMTs (1, 3, 4, 28, 29). Given the varying severity of COVID-19 and the rapid spread of SARS-CoV-2, vaccine-mediated protection of vulnerable people, e.g., pwMS treated with different DMTs, has gained particular attention. Determining the optimal vaccination regimen for those patients remains challenging, especially for pwMS receiving Bc-mts. In this context, studies assessing the benefit of a COVID-19 booster vaccination on the SARS-CoV-2 specific immune response yielded controversial results (7–10, 12). Analyzing anti-SARS-CoV-2(S)-antibody titers after the second, third, and fourth vaccine in OCR-pwMS, we found a significant increase in antibody levels (pv2: 30.4%, pv3: 56.5%, pv4: 90.0%). Likewise, the percentage of OCR-pwMS with positive anti-SARS-CoV-2(S)-antibody titers was higher pv3 compared to pv2 and pv4 compared to pv3, respectively. More than one third of OCR-pwMS with negative anti-SARS-CoV-2(S)-antibodies after two vaccinations had detectable antibodies after the third vaccine dose, which is in line with previous data (11) and validates that COVID-19 booster vaccination increases antibody titers. In general, it is surprising that patients receiving B cell depleting therapy are still able to mount an antibody response. This could be due to the fact that i) usually not 100% of CD20-expressing B cells are depleted and that ii) the CD20-negative B cell compartment also contributes to antibody production. In accordance with previous observations (1, 3, 4, 30), anti-SARS-CoV-2(S)-antibodies positively correlated with peripheral B cell counts as well as with the time between the first vaccination and the last OCR cycle. Mechanistically, B cell progenitors differentiate to plasmablasts and plasma cells upon antigen stimulation which, in turn, produce antigen-specific immunoglobulins (31, 32). The negative correlation between anti-SARS-CoV-2(S)-antibodies and the number of previous OCR cycles underscores the long-lasting immunomodulation mediated by Bc-mts (33). The relevance of our findings regarding the clinical protection of pwMS from SARS-CoV-2 infection and COVID-19 is particularly important for clinical practice. In pwMS receiving Bc-mts, it may help to optimize vaccination schemes and patient monitoring. Although pwMS treated with Bc-mts and S1P-modulators have an attenuated humoral and/or T cellular immune response, our data did not point towards a higher probability of SARS-CoV-2 infection or towards a more severe disease course among such patients. In fact, the amount of asymptomatic and symptomatic SARS-CoV-2 infections was comparable between pwMS on different DMTs, pwMS w/o DMTs, and HC. Of note, none of the pwMS experienced a severe COVID-19 disease course requiring hospitalization or ICU treatment. This indicates effective vaccine-mediated clinical protection of pwMS irrespective of treatment regimen. However, given the low patient numbers, it seems a bit premature to draw definitive conclusions from our cohort. Multicenter studies will be required to address this question. Furthermore, it is conceivable that our results were influenced by differences in protective behavior between cohorts, which should also be addressed in future studies.

In addition, in-depth analysis could not identify significant differences in anti-SARS-CoV-2(S)-antibody or SARS-CoV-2-specific T cell response pv2 between patients who became infected with SARS-CoV-2 and non-infected patients. Thus, even OCR-pwMS who were not able to mount a sufficient humoral immune response following SARS-CoV-2 vaccination were effectively protected from severe COVID-19. Taking into account the preserved SARS-CoV-2-specific T cell response in nearly all OCR-pwMS, this might be the result of a compensatory SARS-CoV-2-specific T cell response as described previously (1). In this regard, comparison of abs^-^IGRA^+^- and abs^+^IGRA^+^-OCR-pwMS revealed similar risks of SARS-CoV-2 infection and symptomatic COVID-19, further corroborating this assumption. Correspondingly, previous studies emphasize the importance of a robust T cell response for clinical protection, especially from severe COVID-19 disease courses (34). Accordingly, in an animal model, T cells mediated effective clinical protection from COVID-19 even in the absence of an antibody response (35).

Furthermore, we found that non-infected pwMS had received significantly more vaccinations compared to patients who were infected with SARS-CoV-2 or suffered from COVID-19. This emphasizes the positive effects of COVID-19 booster vaccinations even in pwMS who are not able to mount a sufficient SARS-CoV-2-specific humoral immune response. This is in concordance with a previous study reporting a significant reduction in SARS-CoV-2 infection after the third vaccine dose (36). Interestingly, pwMS infected with SARS-CoV-2 tended to be younger than non-infected patients. In the general population, the risk for SARS-CoV-2 infection seems to be similar among different age groups. Nevertheless, the risk for hospitalization and death due to COVID-19 significantly increases with age (37). In our cohort of pwMS, none of the patients experienced a severe COVID-19 disease course. This might be due to a generally more protective behavior among those patients, especially older pwMS. Apart from the differences in age between infected and non-infected patients, we found more female than male OCR-pwMS to be infected with SARS-CoV-2. Gender aspects in the COVID-19 pandemic have been previously assessed (38, 39). In this context, a higher infection risk was observed among women at working age, which was attributed to differences in social behavior with women having a higher number of contacts (38). In contrast, male sex was identified as a risk factor for death due to COVID-19 (39). Extending our knowledge on gender aspects is important for optimal prevention and treatment of diseases as for example COVID-19. Regarding the safety profile of COVID-19 vaccines, the short-term safety profile seems to be favorable among pwMS as revealed by our own study and previous ones (1, 40, 41). Even one year after the first vaccination, no relevant side effects were observed in our cohort demonstrating the safety of COVID-19 vaccines for pwMS. Side effects were even less pronounced in pwMS compared to HC, which might be due to the differences in age between groups as correlation analysis revealed a negative correlation between the number of side effects and age. Increased tolerability of SARS-CoV-2 vaccines in the elderly population has been previously described (42). Changes in the immune response in the sense of immunosenescence might contribute to this observation (42).

With regard to disease activity, in two patients, the first MS relapse leading to an eventual diagnosis of RRMS occurred in close temporal association with COVID-19 vaccination. In addition, one RRMS patient without DMT reported MS symptoms fulfilling the criteria for a relapse. However, given the high prevalence of RRMS within the population, the natural relapsing-remitting disease course, and the absence of relapses in pwMS receiving DMTs, an association between COVID-19 vaccination and MS relapses seems unlikely. Although relapses in association with vaccination have been reported (43, 44), a prospective, multicentric observational study could not find an increased short-term risk of clinical relapses after mRNA COVID-19 vaccination (41). However, further large prospective long-term studies are necessary to clarify this issue.

We acknowledge that our study is limited by its retrospective design and the high variability in time between vaccination and anti-SARS-CoV-2(S)-antibody testing. This was primarily due to data acquisition during routinely scheduled clinical workups. It is therefore conceivable that weak antibody responses following vaccination have not been detected, especially in patients with long latencies between analysis of anti-SARS-CoV-2(S)-antibodies and last vaccination. Furthermore, no data on anti-SARS-CoV-2(S)-antibody titers were available for the control groups and no information on the SARS-CoV-2-variants were available, which might impact disease severity. Another limitation of the study was that the type of vaccine was not captured in all cases. Patients received vaccines from various companies. Thus, the overall patient number in every subgroup would have been too small to perform a meaningful statistical analysis regarding vaccine-type associated effects. On the other hand, the large cohort of pwMS receiving different DMTs, the inclusion of HC, and the analysis of the humoral and T cellular vaccine-induced immune response in combination with SARS-CoV-2 infection status and COVID-19 disease course are the main strengths of our study. Regarding potential bias, we did not subselect OCR-pwMS despite reasonable exclusion criteria (e.g., based on EDSS, disease course, disease duration, number of previous OCR cycles). In addition, the same methods were used for all pwMS as well as for HC and negative results were included in the manuscript.

In conclusion, additional COVID-19 vaccinations can boost the humoral immune response in OCR-pwMS and are associated with improved clinical protection against SARS-CoV-2. COVID-19 vaccines mediate effective clinical protection of OCR-pwMS irrespective of the anti-SARS-CoV-2(S)-antibody status indicating compensatory, e.g., T cell mediated, immunological mechanisms.

## Data availability statement

The raw data supporting the conclusions of this article will be made available by the authors, without undue reservation.

## Ethics statement

The studies involving human participants were reviewed and approved by Ethics Comittee, Faculty of Medicine, Heinrich Heine University Düsseldorf, Düsseldorf, Germany. The patients/participants provided their written informed consent to participate in this study.

## Author contributions

SR, AW, MK, TK, NH, KG, BP, JS, CS, CN, LR-N, MF, MR identified patients. SR, AW, MK, TK, NH, KG, BP, JS, CS, CN, LR-N, MR performed data acquisition. SR performed data analyses. SM and DK conceived the study. SM and DK supervised the study. NM, TR, OA, and PA co-supervised the study. SR, AW and DK wrote the manuscript. MB and H-PH gave valuable scientific input to the manuscript. All authors contributed to the article and approved the submitted version.
