# Supplementary material for: Vaccine-based clinical protection against SARS-CoV-2 infection and the humoral immune response: A 1-year follow-up study of patients with multiple sclerosis receiving ocrelizumab
Source: Front Immunol. 2022 Dec 23;13:1037214. doi: 10.3389/fimmu.2022.1037214 (PMC9822773; doi:10.3389/fimmu.2022.1037214)
Supplement: Supplementary file 1 [file DataSheet_1.pdf]

Supplementary figure 1

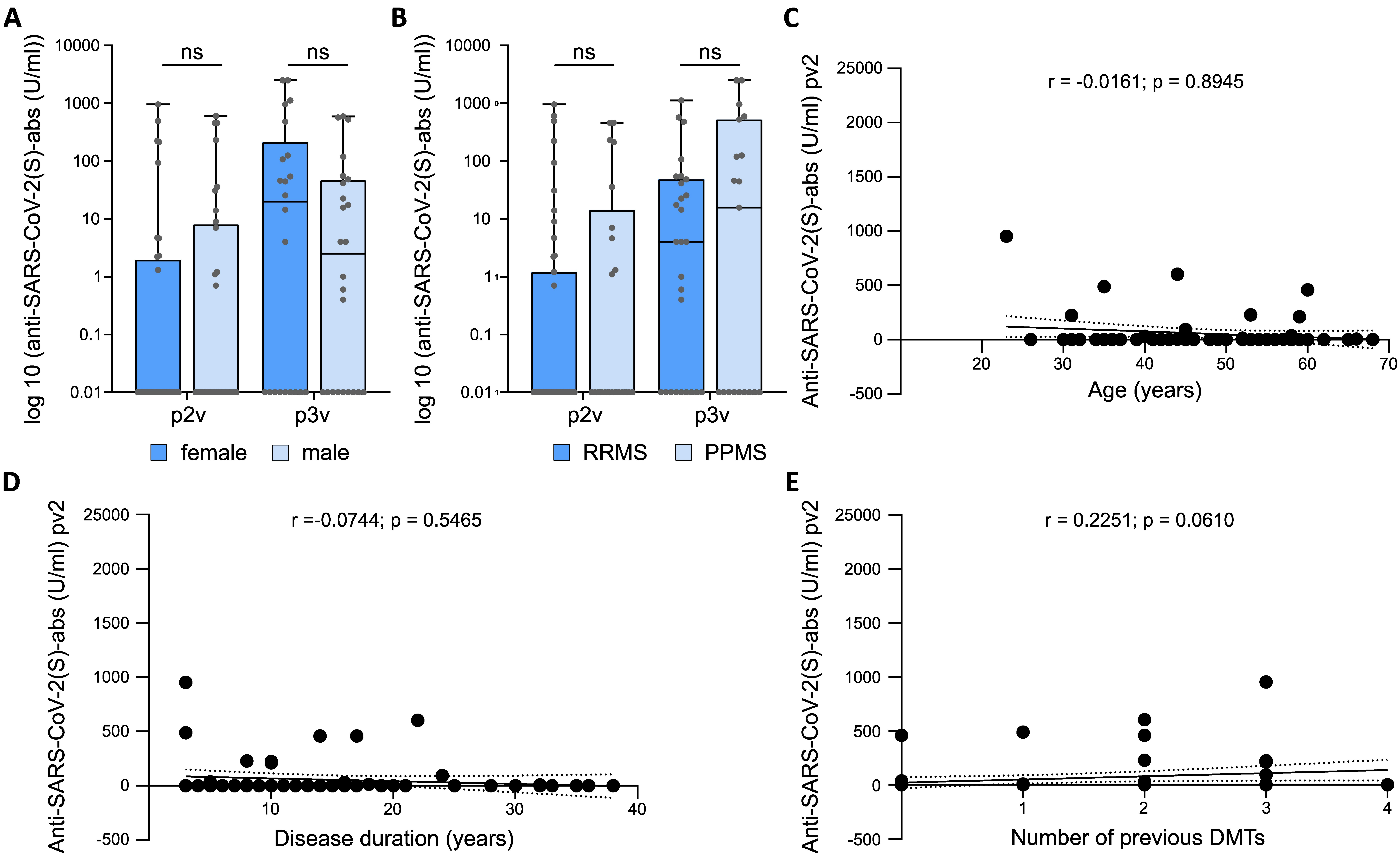

Supplementary figure 1: Additional analyses of anti-SARS-CoV-2-antibodies pv2 and pv3 in patients with MS receiving ocrelizumab

Comparison of anti-SARS-CoV-2(S)-antibodies between female and male OCR-pwMS (**A**) as well as between RRMS and PPMS OCR-pwMS (**B**). Values < 0.4 U/ml are depicted on the x-axis. Correlation analyses of anti-SARS-CoV-2(S)-antibodies with age (**C**), disease duration (**D**), and number of previous DMTs (**E**). Correlation analyses were performed with the Spearman correlation coefficient. The area in-between the dotted lines shows the 95% confidence interval.

Abs: antibodies; DMTs: disease-modifying therapies; ns: not significant; OCR: ocrelizumab; PPMS: primary progressive multiple sclerosis; pv2/3: post second/third vaccination; RRMS: relapsing-remitting multiple sclerosis; S: spike; SARS-CoV-2: severe acute respiratory syndrome.

Supplementary figure 2

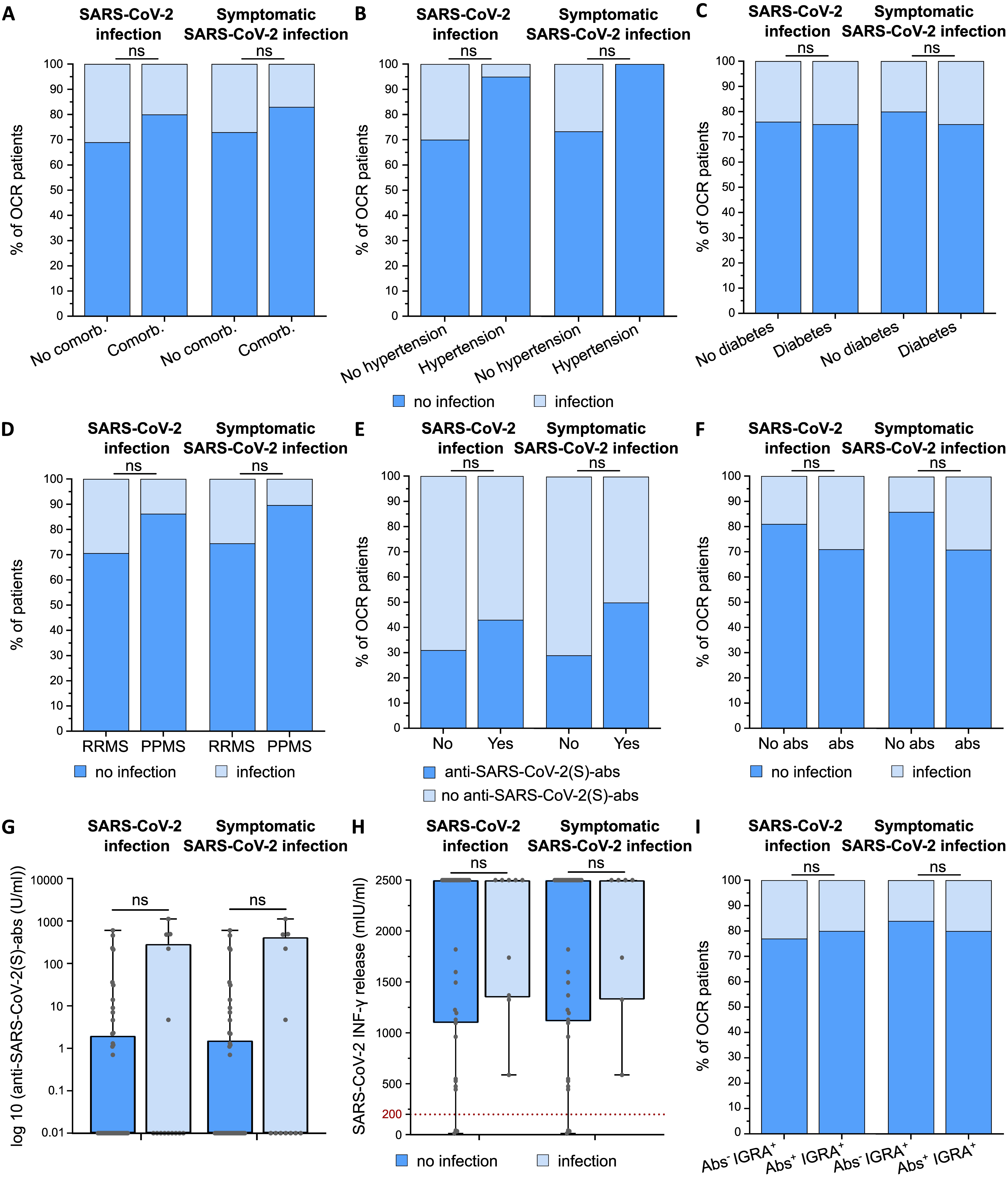

Supplementary figure 2: Additional analyses of SARS-CoV-2 infected compared to non-infected MS patients treated with ocrelizumab

Comparison of comorbidities in general (**A**), hypertension (**B**), and diabetes (**C**) between SARS-CoV-2 infected versus non-infected as well as OCR-pwMS with and without symptomatic COVID-19; **D**: Differences in the probability of SARS-CoV-2 infection between RRMS and PPMS patients; **E** Differences in percentage of patients with positive anti-SARS-CoV-2(S)-antibodies pv2 between infected and non-infected OCR-pwMS as well as patients with and without symptomatic COVID-19; Comparison of anti-SARS-CoV-2(S)-antibody titers (**F**) and INF-γ release by SARS-CoV-2-specific T cells (**G**) between infected and non-infected OCR-pwMS as well as patients with and without symptomatic COVID-19; (**I**) Differences in the probability of SARS-CoV-2 infection between seronegative OCR-pwMS with a detectable SARS-CoV-2-specific T cell response ($abs^{-}IGRA^{+}$) and seropositive OCR-pwMS with a SARS-CoV-2-specific T cell response ($abs^{+}IGRA^{+}$).

Abs: antibodies; Comorb.: comorbidities; COVID-19: coronavirus disease 2019; IFN: interferon; IGRA: Interferon-γ Release Assay; ns: not significant; OCR: ocrelizumab; PPMS: primary progressive multiple sclerosis; RRMS: relapsing-remitting multiple sclerosis; S: spike; SARS-CoV-2: severe acute respiratory syndrome.

Supplementary table 1

Pseudonym	Group	DMT	Diagnosis	Time since 1. symptoms (y)	Time since diagnosis (y)	Duration of current DMT treatment (y)	No previous DMTs	Previous DMTs	Last DMT	Comorbidities
COV-1000000	OCR	Ocrelizumab	PPMS	5	4	1	0	No	No	Hypertension
COV-7476114	OCR	Ocrelizumab	RRMS	36	27	2	1	Teriflunomide	TFN	Hypertension
COV-4041276	OCR	Ocrelizumab	RRMS	11	9	4	2	Interferon-ß<br>Natalizumab	NAT	Obesity
COV-3420723	OCR	Ocrelizumab	PPMS	12	5	4	0	No	No	Hypertension
COV-1000004	OCR	Ocrelizumab	PPMS	6	5	4	0	No	No	S/p hysterectomy
COV-5397951	OCR	Ocrelizumab	RRMS	13	12	3	4	Interferon-ß<br>Dimethyl fumarate<br>Fingolimod<br>Daclizumab	DAC	No
COV-7557777	OCR	Ocrelizumab	RRMS	3	3	3	1	Dimethyl fumarate	DMF	Diabetes<br>Anisometropia<br>Amblyopia (left eye)<br>Anemia
COV-7157652	OCR	Ocrelizumab	RRMS	7	6	4	1	Dimethyl fumarate	DMF	Depression
COV-7571142	OCR	Ocrelizumab	RRMS	32	21	2	4	Interferon-ß<br>Teriflunomide<br>Dimethyl fumarate<br>Recurrent IVIG	DMF/ IVIG	No
COV-3820545	OCR	Ocrelizumab	RRMS	35	29	4	1	Interferon-ß	IFN	Factor V Leiden<br>S/p DVTs
COV-7219212	OCR	Ocrelizumab	RRMS	8	3	3	0	No	No	Hypertension<br>Vitamin D and B12 deficiency
COV-4371330	OCR	Ocrelizumab	PPMS	19	19	3	1	Fingolimod	FNG	No
COV-4620651	OCR	Ocrelizumab	RRMS	5	5	4	0	No	No	No
COV-6963558	OCR	Ocrelizumab	PPMS	8	5	4	0	No	No	Depression
COV-6831210	OCR	Ocrelizumab	RRMS	6	5	4	0	No	No	Migraine<br>Hypertension
COV-5049255	OCR	Ocrelizumab	RRMS	16	15	3	2	Interferon-ß<br>Natalizumab	NAT	S/p testicular torsion<br>S/p tonsillectomy<br>S/p cavernoma
COV-1000006	OCR	Ocrelizumab	RRMS	17	11	2	2	Dimethyl fumarate<br>Interferon-ß	DMF	Migraine
COV-1000007	OCR	Ocrelizumab	PPMS	33	16	3	0	No	No	No

Supplementary table 1

Pseudonym	Group	DMT	Diagnosis	Time since 1. symptoms (y)	Time since diagnosis (y)	Duration of current DMT treatment (y)	No previous DMTs	Previous DMTs	Last DMT	Comorbidities
COV-7235682	OCR	Ocrelizumab	RRMS	3	2	3	0	No	No	S/p jugular vein thrombosis<br>S/p DVT and PE<br>Hypertension<br>Gastroesophageal reflux
COV-7427208	OCR	Ocrelizumab	PPMS	21	16	3	0	No	No	No
COV-6304611	OCR	Ocrelizumab	PPMS	10	8	3	1	Dimethyl fumarate	DMF	Migraine<br>Hypertension
COV-1000009	OCR	Ocrelizumab	RRMS	16	16	4	3	Glatiramer acetate<br>Interferon-ß<br>Natalizumab	NAT	No
COV-7117359	OCR	Ocrelizumab	RRMS	5	4	4	0	No	No	No
COV-4180068	OCR	Ocrelizumab	RRMS	18	14	3	1	Interferon-ß	IFN	Ischemic heart disease<br>S/p myocardial infarction<br>Hypertension<br>Elevated level of lipoprotein(a)
COV-5054247	OCR	Ocrelizumab	RRMS	33	15	4	1	Natalizumab	NAT	S/p hepatitis B<br>S/p trgeminal neuralgia<br>S/p herpes zoster<br>S/p psychogenic pseudo-syncope
COV-1000011	OCR	Ocrelizumab	PPMS	7	6	4	0	No	No	Iridocyclitis<br>Hashimoto thyroiditis
COV-5886468	OCR	Ocrelizumab	RRMS	10	9	9	0	No	No	Hypertension<br>Hypercholesterolemia
COV-7016889	OCR	Ocrelizumab	PPMS	6	5	5	0	No	No	No
COV-3827556	OCR	Ocrelizumab	RRMS	3	2	1	1	Dimethyl fumarate	DMF	No
COV-4707612	OCR	Ocrelizumab	RRMS	17	16	4	3	Interferon-ß<br>Fingolimod<br>Natalizumab	NAT	No
COV-4110633	OCR	Ocrelizumab	PPMS	6	3	2	0	No	No	Hypertension
COV-3245193	OCR	Ocrelizumab	PPMS	7	5	4	0	No	No	No
COV-4170579	OCR	Ocrelizumab	PPMS	8	7	3	0	No	No	S/p melanoma<br>S/p disc herniation<br>Spinal stenosis<br>Tension headache

Supplementary table 1

Pseudonym	Group	DMT	Diagnosis	Time since 1. symptoms (y)	Time since diagnosis (y)	Duration of current DMT treatment (y)	No previous DMTs	Previous DMTs	Last DMT	Comorbidities
COV-5241627	OCR	Ocrelizumab	PPMS	14	6	4	0	No	No	Hypertension<br>Chronic gastritis<br>S/p herpes zoster
COV-5885262	OCR	Ocrelizumab	PPMS	11	8	4	0	No	No	Epilepsy
COV-7008129	OCR	Ocrelizumab	PPMS	11	8	4	1	Interferon-ß	INF	No
COV-7290648	OCR	Ocrelizumab	PPMS	33	3	3	0	No	No	COPD
COV-4356534	OCR	Ocrelizumab	RRMS	20	15	3	1	Natalizumab	NAT	No
COV-6925857	OCR	Ocrelizumab	PPMS	11	7	3	1	Interferon-ß	INF	RLS<br>S/p thyroidectomy
COV-5646105	OCR	Ocrelizumab	RRMS	16	13	3	2	Interferon-ß<br>Natalizumab	NAT	Hip osteonecrosis
COV-7331280	OCR	Ocrelizumab	RRMS	30	25	3	1	Dimethyl fumarate	DMF	No
COV-3981123	OCR	Ocrelizumab	PPMS	21	13	4	1	Interferon-ß	INF	Tremor<br>Hypothyroidism
COV-1000012	OCR	Ocrelizumab	RRMS	2	2	1	0	No	No	No
COV-1000013	OCR	Ocrelizumab	n/a	n/a	n/a	n/a	n/a	n/a	n/a	n/a
COV-1000014	OCR	Ocrelizumab	RRMS	5	5	2	3	Interferon-ß<br>Natalizumab<br>Fingolimod	FNG	Diabetes<br>Depression<br>Migraine<br>Hyperlipidemia
COV-1000015	OCR	Ocrelizumab	RRMS	8	8	3	2	Interferon-ß<br>Fingolimod	FNG	No
COV-1000016	OCR	Ocrelizumab	RRMS	n/a	16	1	3	Interferon-ß<br>Glatiramer acetate<br>Dimethyl fumarate	DMF	Vitamin D deficiency<br>Sicca syndrome<br>Psoriasis<br>Chronic gastritis<br>Hypothyroidism
COV-1000017	OCR	Ocrelizumab	RRMS	14	13	3	1	Interferon-ß	IFN	Depression<br>S/p hepatitis A in childhood
COV-1000018	OCR	Ocrelizumab	PPMS	7	3	2	0	No	No	Hypertension
COV-1000019	OCR	Ocrelizumab	RRMS	7	7	2	2	Natalizumab<br>Teriflunomide	NAT	Migraine<br>Vitamin B12 deficiency<br>Ventricular septal defect

Supplementary table 1

Pseudonym	Group	DMT	Diagnosis	Time since 1. symptoms (y)	Time since diagnosis (y)	Duration of current DMT treatment (y)	No previous DMTs	Previous DMTs	Last DMT	Comorbidities
COV-1000021	OCR	Ocrelizumab	RRMS	11	8	1	1	Fingolimod	FNG	Migraine<br>Chronic mitral regurgitation<br>Asthma<br>Hypercholesterolemia<br>Osteoarthritis<br>Chronic uveitis
COV-1000022	OCR	Ocrelizumab	RRMS	32	3	3	0	No	No	Hypertension<br>Rosacea
COV-4329933	OCR	Ocrelizumab	PPMS	38	19	4	2	Interferon-ß<br>Dimethyl fumarate	DMF	Hypertension
COV-4585662	OCR	Ocrelizumab	RRMS	5	4	3	4	Interferon-ß<br>Glatiramer acetate<br>Teriflunomide<br>Dimethyl fumarate	DMF	No
COV-7273494	OCR	Ocrelizumab	RRMS	22	20	4	4	Dimethyl fumarate<br>Natalizumab<br>Interferon-ß<br>Glatiramer acetate	DMF	S/p retinal detachment
COV-3633750	OCR	Ocrelizumab	RRMS	25	20	4	2	Interferon-ß<br>Fingolimod	FNG	Hypertension<br>Depression<br>S/p hysterectomy
COV-7443393	OCR	Ocrelizumab	RRMS	4	3	3	0	No	0	Asthma
COV-7297536	OCR	Ocrelizumab	PPMS	6	5	3	0	No	0	Hypertension
COV-1000022	OCR	Ocrelizumab	RRMS	19	16	4	4	Teriflunomide<br>Natalizumab<br>Interferon-ß<br>Rituximab	Ritux	No
COV-1000024	OCR	Ocrelizumab	RRMS	24	3	2	0	No	0	Gastritis
COV-1000025	OCR	Ocrelizumab	RRMS	32	13	1	3	Glatiramer acetate<br>Fingolimod<br>Natalizumab	NAT	Hashimoto thyroiditis<br>Rosacea
COV-1000026	OCR	Ocrelizumab	PPMS	8	5	2	0	No	0	S/p stroke
COV-1000027	OCR	Ocrelizumab	RRMS	19	15	3	1	Mitoxantrone	MITOX	Anemia
COV-1000028	OCR	Ocrelizumab	RRMS	13	13	2	2	Interferon-ß<br>Natalizumab	NAT	No

Supplementary table 1

Pseudonym	Group	DMT	Diagnosis	Time since 1. symptoms (y)	Time since diagnosis (y)	Duration of current DMT treatment (y)	No previous DMTs	Previous DMTs	Last DMT	Comorbidities
COV-1000029	OCR	Ocrelizumab	PPMS	13	12	4	3	Interferon-ß<br>Teriflunomide<br>Natalizumab	NAT	No
COV-1000030	OCR	Ocrelizumab	RRMS	15	14	2	3	Glatiramer acetate<br>Dimethyl fumarate<br>Fingolimod	FNG	Pituitary adenoma<br>Depression<br>Retinal migraine
COV-1000031	OCR	Ocrelizumab	RRMS	12	12	2	2	Interferon-ß<br>Fingolimod	FNG	Ulcerative colitis
COV-1000032	OCR	Ocrelizumab	RRMS	21	21	3	4	Interferon-ß<br>Glatiramer acetate<br>Dimethyl fumarate<br>Natalizumab	DMF	S/p thrombosis
COV-5365248	OCR	Ocrelizumab	RRMS	12	12	7	4	Interferon-ß<br>Fingolimod<br>Natalizumab<br>Daclizumab	DAC	Migraine<br>CTS bilaterally<br>S/p ovarian cystectomy
COV-6246162	OCR	Ocrelizumab	RRMS	14	13	4	3	Interferon-ß<br>Dimethyl fumarate<br>Natalizumab	NAT	No
COV-1000033	OCR	Ocrelizumab	RRMS	9	9	3	1	Dimethyl fumarate	DMF	No
COV-5867352	OCR	Ocrelizumab	RRMS	10	10	2	2	Interferon-ß<br>Natalizumab	NAT	No
COV-4929066	OCR	Ocrelizumab	RRMS	15	14	4	3	Glatiramer acetate<br>Natalizumab<br>Dimethyl fumarate	DMF	Narcolepsy<br>S/p colloid cysts<br>S/p pituitary adenoma<br>Hip dysplasia
COV-4127682	OCR	Ocrelizumab	RRMS	33	31	4	3	Glatiramer acetate<br>Natalizumab<br>Dimethyl fumarate	DMF	No
COV-535458	OCR	Ocrelizumab	RRMS	28	26	4	3	Interferon-ß<br>Glatiramer acetate<br>Fingolimod	GLA	Hypothyroidism
COV-5545341	OCR	Ocrelizumab	RRMS	16	15	3	2	Interferon-ß<br>Natalizumab	NAT	No
COV-6537417	OCR	Ocrelizumab	RRMS	8	4	2	1	Interferon-ß	INF	No
COV-7310349	OCR	Ocrelizumab	PPMS	17	17	4	1	Dimethyl fumarate	DMF	No

Supplementary table 1

Pseudonym	Group	DMT	Diagnosis	Time since 1. symptoms (y)	Time since diagnosis (y)	Duration of current DMT treatment (y)	No previous DMTs	Previous DMTs	Last DMT	Comorbidities
COV-1000035	OCR	Ocrelizumab	PPMS	14	9	5	2	Rituximab<br>Mitoxantrone	MITOX	AVNRT<br>Coronary artery disease<br>Hypertension<br>OSAS
COV-1000036	OCR	Ocrelizumab	RRMS	2	2	2	0	No	No	No
COV-1000037	OCR	Ocrelizumab	RRMS	4	4	3	0	No	No	Hypertension<br>Hypothyroidism
COV-1000038	OCR	Ocrelizumab	RRMS	n/a	37	4	0	No	No	S/p pneumonia<br>Thrombocytopenia<br>S/p C. difficile infection<br>S/p sepsis<br>S/p acute kidney failure<br>S/p seizure<br>Hypertension
COV-1000039	OCR	Ocrelizumab	PPMS	8	2	2	0	No	No	Hypercholesterolemia<br>Hypertension<br>Diabetes<br>S/p thyroidectomy
COV-1000040	OCR	Ocrelizumab	PPMS	8	8	2	1	Teriflunomide	TFN	Depression<br>Polyneuropathy
COV-1000034	OCR	Ocrelizumab	RRMS	9	7	2	2	Natalizumab<br>Dimethyl fumarate	NAT	Depression
COV-1000041	OCR	Ocrelizumab	PPMS	11	11	4	0	No	No	Diabetes<br>Hypertension<br>S/p thyroidectomy
COV-1000042	OCR	Ocrelizumab	PPMS	4	2	1	0	No	No	Depression
COV-1000043	OCR	Ocrelizumab	RRMS	5	4	2	1	Dimethyl fumarate	DMF	No
MS-4	NAT	Natalizumab	RRMS	4	4	2	1	Dimethyl fumarate	DMF	ADHD<br>Strabismus
MS-17	NAT	Natalizumab	RRMS	14	14	13	1	Interferon-ß	IFN	No
MS-21	NAT	Natalizumab	RRMS	14	14	10	1	Glatiramer acetate	GLA	No
MS-25	NAT	Natalizumab	RRMS	5	5	0	3	Glatiramer acetate<br>Dimethyl fumarate<br>Interferon-ß	IFN	No
MS-36	NAT	Natalizumab	RRMS	1	1	0	0	No	No	Latent tuberculosis infection<br>Scalp eczema

Supplementary table 1

Pseudonym	Group	DMT	Diagnosis	Time since 1. symptoms (y)	Time since diagnosis (y)	Duration of current DMT treatment (y)	No previous DMTs	Previous DMTs	Last DMT	Comorbidities
MS-40	NAT	Natalizumab	RRMS	7	7	1	2	Interferon-ß<br>Dimethyl fumarate	DMF	No
MS-43	NAT	Natalizumab	RRMS	26	12	4	1	Interferon-ß	IFN	Hypertension
MS-44	NAT	Natalizumab	RRMS	15	15	14	1	Interferon-ß	IFN	Depression
MS-45	NAT	Natalizumab	RRMS	29	28	14	1	Interferon-ß	IFN	No
MS-51	NAT	Natalizumab	RRMS	19	19	15	1	Interferon-ß	IFN	No
MS-55	NAT	Natalizumab	RRMS	22	18	10	1	Interferon-ß	IFN	Epilepsy
MS-56	NAT	Natalizumab	RRMS	24	24	21	1	Interferon-ß	IFN	Vitamin D deficiency<br>Depression
MS-58	NAT	Natalizumab	RRMS	2	2	1	0	No	No	Migraine
MS-59	NAT	Natalizumab	RRMS	6	2	0	0	No	No	No
MS-64	NAT	Natalizumab	RRMS	16	16	11	1	Interferon-ß	IFN	Diabetes<br>Hypertension
MS-65	NAT	Natalizumab	RRMS	9	8	3	2	Dimethyl fumarate<br>Fingolimod	FNG	Depression
MS-66	NAT	Natalizumab	RRMS	24	19	9	1	Interferon-ß	IFN	No
MS-67	NAT	Natalizumab	RRMS	14	14	2	2	Dimethyl fumarate<br>Fingolimod	FNG	Iron deficiency<br>Vitamin D deficiency
MS-69	NAT	Natalizumab	RRMS	16	16	10	2	Interferon-ß<br>Glatiramer acetate	GLA	No
MS-70	NAT	Natalizumab	RRMS	15	14	4	1	Interferon-ß	IFN	No
MS-74	NAT	Natalizumab	RRMS	16	16	14	1	Fingolimod	FNG	No
MS-78	NAT	Natalizumab	RRMS	20	15	13	1	Interferon-ß	IFN	Diabetes<br>Hypothyroidism
MS-85	NAT	Natalizumab	RRMS	13	12	8	1	Interferon-ß	IFN	No
MS-86	NAT	Natalizumab	RRMS	23	23	8	1	Glatiramer acetate	GLA	No
MS-89	NAT	Natalizumab	RRMS	17	17	10	3	Glatiramer acetate<br>Interferon-ß<br>Azathioprine	AZA	No
MS-94	NAT	Natalizumab	RRMS	2	2	2	0	No	No	Depression
MS-97	NAT	Natalizumab	RRMS	18	18	13	1	Interferon-ß	IFN	Disc herniation
MS-100	NAT	Natalizumab	SPMS	26	26	11	3	Glatiramer acetate<br>Interferon-ß<br>Mitoxantrone	MITOX	No
MS-111	NAT	Natalizumab	RRMS	21	21	15	1	Glatiramer acetate	GLA	Asthma
MS-113	NAT	Natalizumab	RRMS	28	28	13	1	Interferon-ß	IFN	No

Supplementary table 1

Pseudonym	Group	DMT	Diagnosis	Time since 1. symptoms (y)	Time since diagnosis (y)	Duration of current DMT treatment (y)	No previous DMTs	Previous DMTs	Last DMT	Comorbidities
MS-114	NAT	Natalizumab	RRMS	16	16	15	1	Interferon-ß	IFN	Diabetes<br>Hypertension
MS-118	NAT	Natalizumab	RRMS	14	9	6	2	Dimethyl fumarate<br>Interferon-ß	IFN	Hypertension<br>Sleep apnea<br>Migraine<br>Vitamin B6 deficiency
MS-120	NAT	Natalizumab	RRMS	14	14	8	2	Interferon-ß<br>Fingolimod	FNG	No
MS-121	NAT	Natalizumab	RRMS	0	0	0	0	No	No	No
MS-123	NAT	Natalizumab	RRMS	27	24	12	2	Glatiramer acetate<br>Interferon-ß	IFN	No
MS-130	NAT	Natalizumab	RRMS	4	4	3	1	Dimethyl fumarate	DMF	No
MS-145	NAT	Natalizumab	RRMS	8	8	5	4	Dimethyl fumarate<br>Interferon-ß<br>Glatiramer acetate<br>Daclizumab	DAC	No
MS-146	NAT	Natalizumab	RRMS	5	5	2	1	Glatiramer acetate	GLA	No
MS-147	NAT	Natalizumab	RRMS	19	12	10	0	None	No	Migraine
MS-157	NAT	Natalizumab	RRMS	12	8	8	0	None	No	No
MS-158	NAT	Natalizumab	RRMS	6	6	1	2	Interferon-ß<br>Glatiramer acetate	GLA	RLS
MS-160	NAT	Natalizumab	RRMS	18	18	16	2	Interferon-ß<br>Dimethyl fumarate	DMF	No
MS-6	S1P	Fingolimod	RRMS	2	2	1	1	Interferon-ß	IFN	AV block
MS-10	S1P	Siponimod	SPMS	17	17	1	1	Mitoxantrone	MITOX	CKD
MS-18	S1P	Fingolimod	SPMS	24	24	4	1	Interferon-ß	IFN	Hypertension
MS-33	S1P	Fingolimod	RRMS	19	19	7	1	Natalizumab	NAT	Depression<br>Osteoarthritis
MS-37	S1P	Fingolimod	SPMS	17	16	4	1	Interferon-ß	IFN	Hypercholesterolemia<br>Scheuermann kyphosis<br>Hypothyroidism
MS-38	S1P	Fingolimod	SPMS	n/a	12	5	1	Glatiramer acetate	GLA	Anemia
MS-49	S1P	Siponimod	SPMS	15	15	0	3	Fingolimod<br>Glatiramer acetate<br>Cladribine	CLAD	Hypertension

Supplementary table 1

Pseudonym	Group	DMT	Diagnosis	Time since 1. symptoms (y)	Time since diagnosis (y)	Duration of current DMT treatment (y)	No previous DMTs	Previous DMTs	Last DMT	Comorbidities
MS-19	S1P	Fingolimod	RRMS	34	n/a	3	2	Natalizumab<br>Interferon-ß	NAT	OSAS
MS-76	S1P	Fingolimod	RRMS	12	12	3	2	Interferon-ß<br>Glatiramer acetate	GLA	Psoriasis arthritis<br>Hypothyroidism<br>S/p cervical cancer<br>CTS
MS-77	S1P	Ozanimod	RRMS	3	2	1	2	Interferon-ß<br>Glatiramer acetate	GLA	No
MS-83	S1P	Fingolimod	RRMS	13	12	11	1	Interferon-ß	IFN	S/p subarachnoid hemorrhage<br>S/p anorexia nervosa<br>S/p melanoma
MS-90	S1P	Siponimod	SPMS	29	28	1	4	Glatiramer acetate<br>Interferon-ß<br>Azathioprine<br>Natalizumab	NAT	Hypothyroidism<br>Lactose intolerance
MS-95	S1P	Fingolimod	RRMS	5	5	5	1	Interferon-ß	IFN	Hypertension
MS-102	S1P	Fingolimod	RRMS	7	7	4	1	Interferon-ß	IFN	No
MS-106	S1P	Ozanimod	RRMS	22	12	1	4	Glatiramer acetate<br>Interferon-ß<br>Dimethyl fumarate<br>Cladribine	CLAD	Hypothyroidism<br>Migraine
MS-125	S1P	Siponimod	SPMS	8	8	1	2	Interferon-ß<br>Natalizumab	NAT	No
MS-131	S1P	Siponimod	SPMS	41	19	1	1	Dimethyl fumarate	DMF	Hyperthyroidism
MS-137	S1P	Fingolimod	RRMS	9	6	5	1	Dimethyl fumarate	DMF	Autoimmune thyroiditis
MS-139	S1P	Siponimod	SPMS	31	31	1	2	Glatiramer acetat<br>Fingolimod	FNG	S/p breast cancer<br>Hypothyroidism<br>Anemia
MS-144	S1P	Siponimod	SPMS	21	19	1	1	Mitoxantrone	MITOX	Depression<br>S/p breast cancer
MS-148	S1P	Fingolimod	RRMS	32	32	7	2	Interferon-ß<br>Glatiramer acetate	GLA	Asthma<br>Diabetes
MS-155	S1P	Siponimod	SPMS	39	28	1	0	None	No	No
MS-161	S1P	Siponimod	SPMS	19	18	2	3	Interferon-ß<br>Mitoxantrone<br>Ocrelizumab	OCR	No

Supplementary table 1

Pseudonym	Group	DMT	Diagnosis	Time since 1. symptoms (y)	Time since diagnosis (y)	Duration of current DMT treatment (y)	No previous DMTs	Previous DMTs	Last DMT	Comorbidities
MS-162	S1P	Siponimod	SPMS	15	12	1	1	Interferon-ß	IFN	Depression
MS-164	S1P	Fingolimod	RRMS	2	2	2	0	No	No	Vitamin D deficiency
MS-165	S1P	Siponimod	SPMS	19	18	10	2	Fingolimod<br>Interferon-ß	FNG	Hypertension<br>Tricuspid regurgitation<br>Hypercholesterolemia
MS-166	S1P	Fingolimod	RRMS	1	1	1	0	No	No	No
MS-3	No DMT	No	RRMS	7	6	-	1	Galtiramer acetate	GLA	Depression<br>Fibromyalgie<br>Lactose intolerance
MS-7	No DMT	No	RRMS	5	5	-	0	No	No	Epilepsy<br>Ankylosing spondylitis<br>RLS
MS-8	No DMT	No	RRMS	0	0	-	0	No	No	Lipedema<br>Hypertension<br>Asthma<br>Pineal cysts<br>Chronic bronchitis
MS-11	No DMT	No	RRMS	19	0	-	0	No	No	No
MS-12	No DMT	No	RRMS	3	3	-	0	No	No	Hypothyroidism
MS-13	No DMT	No	RRMS	0	0	-	0	No	No	Depression<br>COPD<br>Osteoarthritis
MS-15	No DMT	No	RRMS	12	12	-	2	Fingolimod<br>Teriflunomide	FNG	No
MS-16	No DMT	No	RRMS	10	2	-	1	Teriflunomide	TFN	Hand eczema<br>Hip dysplasia<br>Migraine<br>Asthma<br>Somatic symptom disorder<br>S/p bulimia<br>Hypothyroidism
MS-22	No DMT	No	RRMS	1	0	-	0	No	No	Depression<br>Anxiety disorder
MS-26	No DMT	No	PPMS	3	0	-	0	No	No	No
MS-27	No DMT	No	RRMS	0	0	-	0	No	No	No
MS-28	No DMT	No	RRMS	19	19	-	0	No	No	Vitiligo

Supplementary table 1

Pseudonym	Group	DMT	Diagnosis	Time since 1. symptoms (y)	Time since diagnosis (y)	Duration of current DMT treatment (y)	No previous DMTs	Previous DMTs	Last DMT	Comorbidities
MS-29	No DMT	No	RRMS	4	4	-	0	No	No	Asthma
MS-31	No DMT	No	RRMS	1	0	-	0	No	No	No
MS-32	No DMT	No	RRMS	0	0	-	0	No	No	COPD<br>Polyneuropathy<br>Cataract
MS-35	No DMT	No	PPMS	16	0	-	0	No	No	No
MS-39	No DMT	No	RRMS	n/a	0	-	0	No	No	Bladder cancer<br>Hypercholesterolemia
MS-41	No DMT	No	SPMS	11	11	-	2	Glatiramer acetate<br>Interferon-ß	GLA	Polyneuropathy<br>Diabetes<br>Hypertension<br>Arrythmia
MS-42	No DMT	No	RRMS	1	0	-	0	No	No	No
MS-46	No DMT	No	RRMS	23	3	-	2	Dimethyl fumarate<br>Glatiramer acetate	GLA	Hypertension<br>Glaucoma<br>Hyperthyroidism<br>Graves ophthalmopathy
MS-47	No DMT	No	RRMS	0	0	-	0	No	No	No
MS-48	No DMT	No	RRMS	1	0	-	0	No	No	Hypertension
MS-50	No DMT	No	PPMS	9	0	-	0	No	No	Brest cancer<br>Vitamin B12 deficiency
MS-54	No DMT	No	RRMS	0	0	-	0	No	No	Hip osteoarthritis<br>Alcohol use disorder<br>Steatosis hepatis<br>Pancreatic lipomatosis
MS-57	No DMT	No	RRMS	1	1	-	2	Interferon-ß<br>Glatiramer acetate	GLA	Hypertension<br>Depression<br>Hypothyroidism
MS-62	No DMT	No	PPMS		0	-	0	No	No	No
MS-71	No DMT	No	RRMS	1	1	-	0	No	No	Epilepsy
MS-73	No DMT	No	PPMS	11	9	-	2	Ocrelizumab<br>Fingolimod	OCR	No
MS-75	No DMT	No	RRMS	20	20	-	2	Interferon-ß<br>Glatiramer acetate	n/a	Neurodermatitis
MS-79	No DMT	No	RRMS	12	12	-	1	Dimethyl fumarate	DMF	No
MS-82	No DMT	No	PPMS	27	2	-	0	No	No	Depression

Supplementary table 1

Pseudonym	Group	DMT	Diagnosis	Time since 1. symptoms (y)	Time since diagnosis (y)	Duration of current DMT treatment (y)	No previous DMTs	Previous DMTs	Last DMT	Comorbidities
MS-87	No DMT	No	RRMS	19	17	-	2	Fingolimod<br>Dimethyl fumarate	DMF	S/p disc herniation
MS-91	No DMT	No	RRMS	0	0	-	0	No	No	No
MS-92	No DMT	No	RRMS	2	2	-	0	No	No	Lymphedema<br>Hypertension<br>Rosacea
MS-98	No DMT	No	RRMS	9	9	-	1	Interferon-ß	IFN	Cardiomyopathy<br>Heart failure<br>Hypothyroidism<br>S/p peroneal nerve palsy<br>Latent tuberculosis infection
MS-103	No DMT	No	SPMS	21	21	-	2	Glatiramer acetate<br>Interferon-ß	IFN	Atypical HUS<br>Melanoma<br>Hypertension<br>Chronic bronchitis<br>Neurodermitis<br>S/p acute kidney failure
MS-104	No DMT	No	SPMS	30	30	-	0	No	No	Obsessive-compulsive disorder
MS-108	No DMT	No	PPMS	19	17	-	0	No	No	Hypertension
MS-110	No DMT	No	RRMS	1	0	-	0	No	No	Hypothyroidism<br>Ankylosing spondylitis
MS-115	No DMT	No	PPMS	29	29	-	2	Glatiramer acetate<br>Interferon-ß	GLA	Epilepsy
MS-116	No DMT	No	RRMS	6	0	-	0	No	No	Asthma<br>Hypertension<br>Disc herniation<br>Polyneuropathy
MS-119	No DMT	No	RRMS	9	9	-	2	Glatiramer acetate<br>Fingolimod	FNG	Temporal lobe epilepsy
MS-122	No DMT	No	RRMS	1	0	-	0	No	No	S/p depressive episode
MS-124	No DMT	No	RRMS	0	0	-	0	No	No	No
MS-128	No DMT	No	RRMS	0	0	-	0	No	No	No
MS-129	No DMT	No	RRMS	0	0	-	0	No	No	Asthma
MS-133	No DMT	No	PPMS	8	0	-	0	No	No	Hypertension<br>GERD<br>Spinal stenosis

Supplementary table 1

Pseudonym	Group	DMT	Diagnosis	Time since 1. symptoms (y)	Time since diagnosis (y)	Duration of current DMT treatment (y)	No previous DMTs	Previous DMTs	Last DMT	Comorbidities
MS-134	No DMT	No	RRMS	4	0	-	0	No	No	Subclavian artery stenosis<br>Vitamin D deficiency<br>Hypercholesterolemia<br>Adrenal adenoma
MS-72	No DMT	No	RRMS	0	0	-	0	No	No	No
MS-135	No DMT	No	SPMS	22	22	-	3	Azathioprine<br>Mitoxantrone<br>Interferon-ß	n/a	No
MS-136	No DMT	No	PPMS	4	0	-	0	No	No	No
MS-138	No DMT	No	SPMS	27	12	-	1	Interferon-ß	IFN	No
MS-142	No DMT	No	RRMS	12	12	-	0	No	No	Migraine
MS-143	No DMT	No	RRMS	0	0	-	0	No	No	Breast cancer<br>Migraine<br>S/p glaucoma
MS-149	No DMT	No	SPMS	40	40	-	0	No	No	Hypertension<br>Hypothyroidism<br>GERD<br>Colorectal adenoma
MS-150	No DMT	No	RRMS	0	0	-	0	No	No	Migraine
MS-156	No DMT	No	RRMS	7	1	-	0	No	No	Factor V Leiden
MS-159	No DMT	No	PPMS	11	11	-	2	Teriflunomide<br>Interferon-ß	TFN	Polyneuropathy<br>Diabetes<br>Depression
MS-163	No DMT	No	SPMS	20	20	-	0	No	No	Chronic tension hedeache<br>Hypothyroidism<br>S/p CTS<br>Benign prostatic hyperplasia
HC-11	HC	No	control	-	-	-	0	No	No	No
HC-12	HC	No	control	-	-	-	0	No	No	No
HC-13	HC	No	control	-	-	-	0	No	No	No
HC-14	HC	No	control	-	-	-	0	No	No	No
HC-15	HC	No	control	-	-	-	0	No	No	Migraine<br>Plasmacytoma
HC-17	HC	No	control	-	-	-	0	No	No	No
HC-9	HC	No	control	-	-	-	0	No	No	No
HC-32	HC	No	control	-	-	-	0	No	No	No

Supplementary table 1

Pseudonym	Group	DMT	Diagnosis	Time since 1. symptoms (y)	Time since diagnosis (y)	Duration of current DMT treatment (y)	No previous DMTs	Previous DMTs	Last DMT	Comorbidities
HC-19	HC	No	control	-	-	-	0	No	No	No
HC-21	HC	No	control	-	-	-	0	No	No	No
HC-2	HC	No	control	-	-	-	0	No	No	Chronic sinusitis
HC-3	HC	No	control	-	-	-	0	No	No	Varicosos<br>S/p DVT
HC-4	HC	No	control	-	-	-	0	No	No	No
HC-22	HC	No	control	-	-	-	0	No	No	No
HC-30	HC	No	control	-	-	-	0	No	No	No
HC-7	HC	No	control	-	-	-	0	No	No	No
HC-8	HC	No	control	-	-	-	0	No	No	No
HC-1	HC	No	control	-	-	-	0	No	No	No
HC-40	HC	No	control	-	-	-	0	No	No	No
HC-5	HC	No	control	-	-	-	0	No	No	No
HC-6	HC	No	control	-	-	-	0	No	No	No
HC-33	HC	No	control	-	-	-	0	No	No	Hypertension<br>Osteoarthritis
HC-34	HC	No	control	-	-	-	0	No	No	No
HC-35	HC	No	control	-	-	-	0	No	No	No
HC-36	HC	No	control	-	-	-	0	No	No	No
HC-38	HC	No	control	-	-	-	0	No	No	No
HC-39	HC	No	control	-	-	-	0	No	No	No
HC-10	HC	No	control	-	-	-	0	No	No	No
HC-24	HC	No	control	-	-	-	0	No	No	No
HC-25	HC	No	control	-	-	-	0	No	No	Asthma
HC-26	HC	No	control	-	-	-	0	No	No	Ulcerative colitis
HC-27	HC	No	control	-	-	-	0	No	No	No
HC-28	HC	No	control	-	-	-	0	No	No	No
HC-29	HC	No	control	-	-	-	0	No	No	Allergic rhinitis
HC-31	HC	No	control	-	-	-	0	No	No	No
HC-41	HC	No	control	-	-	-	0	No	No	Asthma
HC-42	HC	No	control	-	-	-	0	No	No	No
HC-43	HC	No	control	-	-	-	0	No	No	No
HC-44	HC	No	control	-	-	-	0	No	No	No
HC-45	HC	No	control	-	-	-	0	No	No	No
HC-46	HC	No	control	-	-	-	0	No	No	Chronic autoimmune thyroiditis
HC-47	HC	No	control	-	-	-	0	No	No	Psoriasis

Supplementary table 1

Pseudonym	Group	DMT	Diagnosis	Time since 1. symptoms (y)	Time since diagnosis (y)	Duration of current DMT treatment (y)	No previous DMTs	Previous DMTs	Last DMT	Comorbidities
HC-48	HC	No	control	-	-	-	0	No	No	Diabetes<br>Hypertension
HC-49	HC	No	control	-	-	-	0	No	No	No
HC-50	HC	No	control	-	-	-	0	No	No	Hypertension
HC-51	HC	No	control	-	-	-	0	No	No	No
HC-52	HC	No	control	-	-	-	0	No	No	No
HC-53	HC	No	control	-	-	-	0	No	No	Polycystic ovary syndrome<br>Autoimmune thyreoiditis
HC-54	HC	No	control	-	-	-	0	No	No	No
HC-55	HC	No	control	-	-	-	0	No	No	No
HC-56	HC	No	control	-	-	-	0	No	No	Hypertension<br>Migraine<br>Myotonia congenita
HC-57	HC	No	control	-	-	-	0	No	No	Traumatic peroneal nerve palsy
HC-58	HC	No	control	-	-	-	0	No	No	No
HC-59	HC	No	control	-	-	-	0	No	No	No
HC-60	HC	No	control	-	-	-	0	No	No	Hypertension<br>S/p bladder cancer
HC-61	HC	No	control	-	-	-	0	No	No	No
HC-62	HC	No	control	-	-	-	0	No	No	Asthma
HC-63	HC	No	control	-	-	-	0	No	No	No
HC-64	HC	No	control	-	-	-	0	No	No	Idiopathic intracranial hypertension
HC-65	HC	No	control	-	-	-	0	No	No	No
HC-66	HC	No	control	-	-	-	0	No	No	Gilbert syndrome

Supplementary table 1: Basic characteristics of patients with MS and controls

ADHD: attention deficit hyperactivity disorder; AV: atrioventricular; AVNRT: AV nodal reentrant tachycardia; AZA: azathioprine; C: clostridium; CKD: chronic kidney disease; CLAD: cladribine; COPD: chronic obstructive pulmonary disease; CTS: carpal tunnel syndrome; DAC: daclizumab; DMF: dimethyl fumarate; DMT: disease modifying therapy; DVT: deep vein thrombosis; f: female; FNG: fingolimod; GERD: gastroesophageal reflux disease; GLA: glatiramer acetate; HUS: hemolytic uremic syndrome; IFN: interferon; IVIG: intravenous immunoglobulins; m: male; MS: multiple sclerosis; n/a: not available; No: number of; MITOX: mitoxantrone; NAT: natalizumab; OCR: ocrelizumab; OSAS: obstructive sleep apnea syndrome; PE: pulmonary embolism; PPMS: primary progressive MS; Ritux: Rituximab; RLS: restless legs syndrome; RRMS: relapsing-remitting MS; SPMS: secondary progressive MS; S/p: status post; TFN: teriflunomide; y: years.
